# Latex Anaphylaxis Caused by Occupational Exposure to Balloons

**DOI:** 10.7759/cureus.25875

**Published:** 2022-06-12

**Authors:** Robert W West, Akbar Sharip

**Affiliations:** 1 Occupational Medicine, Loma Linda University Medical Center, Loma Linda, USA

**Keywords:** latex-free, allergy, latex, anaphylaxis, balloon, occupational, latex allergy

## Abstract

Latex allergies often develop by sensitization to latex allergens by repeated exposure. Because in recent years latex has been ubiquitous in medical equipment, health workers have a higher prevalence of latex allergies than the general population, and care must be taken to ensure workers' safety. We report a case of a female health care worker in her 20s who experienced a severe, biphasic anaphylactic reaction within minutes after being exposed to rubber balloons at a latex-free children’s hospital. After being stabilized with epinephrine, dexamethasone, and fluid resuscitation, over a six-hour period, she was discharged home. En route home, her symptoms recurred, and she was admitted to the ICU for observation for impending respiratory failure. She was hospitalized for about 48 hours before being discharged home. She presented to the occupational medicine clinic a few days later for further management. No acute care was required and she was discharged.

This case is consistent with occupational latex-induced anaphylaxis. Health personnel should be educated about the importance of compliance with latex allergy mitigation procedures, as well as the severe nature of hypersensitivity reactions that may occur in sensitized persons. It may be beneficial to address the social pressures that can contribute to noncompliance, as balloons are a common gift for children and may be viewed as an acceptable way to cheer up a sick child, tempting some staff to turn a blind eye to policy. The reasons for the policy, and for strict adherence, should be communicated clearly.

## Introduction

Latex allergy is a common occupational health hazard. The health care profession is at particularly high risk. Latex allergy is usually the result of sensitization over a long term period, and the ubiquity of latex materials in health care in the early 1990s prompted a spike in latex allergy peaking in that decade, but it has fallen recently as non-latex synthetics such as nitrile have come into more widespread use [1]. Still, data suggests that the prevalence of latex allergy may be as high as 9.7% in health care workers worldwide, as recently as 2016 [2].

The most important risk factors for latex allergy among health care workers are occupational exposure and atopy [3]. In susceptible workers, latex allergies can be divided broadly into two classes: allergic contact dermatitis, which is a delayed-type (type iv) hypersensitivity reaction, and immediate, IgE-mediated (type i) hypersensitivity reaction. While both types may result in disabling morbidities, type I hypersensitivities carry the additional risk of lethal anaphylactic reactions. Such reactions are most likely to occur in sensitized individuals in whom exposure occurs rapidly through mucous membranes, such as in the respiratory, gastrointestinal, or reproductive tracts.

## Case presentation

Our patient was a young female in her mid-20s, with a history of atopy, food allergies, and a known allergy to latex, who works as a Patient Care Assistant at a children’s hospital in the United States that has a latex-free facility policy. She had known multiple allergies including pineapple, shellfish, and latex, and has a history of multiple anaphylactic reactions; her most recent episode of anaphylaxis was approximately three weeks prior to the current episode and resulted from likely exposure to pineapple. She carries an EpiPen with her at all times and her family history is non-contributory. She stated that a few days prior to her initial visit to our clinic, she arrived at work to find her ward decorated with balloons in celebration of a patient. She reported being in the same room as the balloons, but not touching nor coming very close to the balloons. 

After a few minutes inside the ward, she acutely developed airway swelling, shortness of breath, cough, and urticaria on her arms. Recognizing symptoms of allergy, she immediately self-administered epinephrine with an EpiPen, took a Benadryl, and went to the emergency department (ED). She reported not staying in the ward with balloons for more than a total of 5-10 minutes.

On arrival at the ED, she was noted to be tachypneic and had wheezing and stridor on exam, as well as a change in voice. A chest X-ray was grossly normal. Complete blood count revealed no abnormalities, and basic metabolic panel (BMP) showed acutely elevated blood glucose of 181. A second dose of intramuscular (IM) epinephrine was given, as well as dexamethasone, famotidine, IV lactated ringers, and two nebulizer treatments with racemic epinephrine. After approximately six hours, she reported good improvement in symptoms and was discharged from the ED.

During her one-hour commute home, she again experienced throat swelling and shortness of breath, and facial tingling. The patient stopped at another hospital ED for treatment. Two additional racemic epinephrine nebulized treatments were administered, but she continued to experience worsening shortness of breath and an IV epinephrine drip was placed. The patient was admitted to the ICU for monitoring of the IV drip and impending respiratory failure. Fortunately, she stabilized in critical care and was able to be transferred to the acute care ward later that day. After approximately 12 hours of additional observation, she was found to be stable and was discharged the next day.

## Discussion

Latex allergy remains a very common and potentially deadly problem among health care workers. Latex sensitization occurs with repeated exposures over time, and as such, health care workers are at exceptionally high risk due to the pervasiveness of this substance in medical equipment [4]. In response to this crisis, in recent years, health care facilities have increasingly adopted latex-free policies to prevent complications of latex allergy. Nonetheless, allergies continue to have a high prevalence in health professionals. 

Health care staff and policymakers should be educated on the dangers of latex allergies. Latex specifically refers to the sap of the Brazilian rubber tree *Hevea brasiliensis*. The raw sap itself produces common latex products such as gloves and condoms, but the sap is refined and coagulated to produce natural rubber, the material used to make such products as tires, balloons, erasers, etc. Importantly, it should be noted that many products not specifically labeled as “latex”, such as rubber products, can therefore still provoke latex allergies [5]. Similarly, the FDA warns that “latex-free” labels may not be accurate and products with this label may nonetheless provoke hypersensitivity reactions in susceptible individuals [6].

The case described demonstrates that health care workers are highly vulnerable to hypersensitivity reactions, even with a seemingly trivial exposure, such as microscopic airborne particles from balloons. The American Academy of Asthma, Allergy, and Immunology warns of severe allergic reactions occurring in sensitized persons simply by being in proximity to balloons in an enclosed space [7].

## Conclusions

Strict adherence to, and enforcement of, latex-free policies is critical in the health care setting; even rare exceptions can jeopardize the wellbeing of patients and staff. It is essential to educate health care employees about latex-free policies, and the hazards associated with violations. Health workers should also be aware of social pressures that are potential obstacles to compliance with latex-free policies; regulations that forbid celebratory balloons to be given to sick children may strike some workers as heartless and excessive, but while the urge to bring joy to sick children is very natural and understandable, even a one-time exception to latex policies can have potentially dire consequences.
